# One year ago not business as usual: Wound management, infection and psychoemotional control during tertiary medical care following the 2004 Tsunami disaster in southeast Asia

**DOI:** 10.1186/cc4868

**Published:** 2006-03-29

**Authors:** Marc Maegele, Sven Gregor, Nedim Yuecel, Christian Simanski, Thomas Paffrath, Dieter Rixen, Markus M Heiss, Claudia Rudroff, Stefan Saad, Walter Perbix, Frank Wappler, Andreas Harzheim, Rosemarie Schwarz, Bertil Bouillon

**Affiliations:** 1 Department of Traumatology and Orthopedic Surgery, Cologne-Merheim Medical Center (CMMC), University of Witten/Herdecke, Ostmerheimerstrasse, 51109 Cologne, Germany; 2 Intensive Care Unit of the Department of Traumatology and Orthopedic Surgery, CMMC, University of Witten/Herdecke, Ostmerheimerstrasse, 51109 Cologne, Germany; 3 Department of Visceral Surgery, CMMC, University of Witten/Herdecke, Ostmerheimerstrasse, 51109 Cologne, Germany; 4 Department of Plastic and Reconstructive Surgery, CMMC, University of Witten/Herdecke, Ostmerheimerstrasse, 51109 Cologne, Germany; 5 Department of Anaesthesiology, CMMC, University of Witten/Herdecke, Ostmerheimerstrasse, 51109 Cologne, Germany; 6 Department of Radiology, CMMC, University of Witten/Herdecke, Ostmerheimerstrasse, 51109 Cologne, Germany; 7 Department of Microbiology, CMMC, University of Witten/Herdecke, Ostmerheimerstrasse, 51109 Cologne, Germany

## Abstract

**Introduction:**

Following the 2004 tsunami disaster in southeast Asia severely injured tourists were repatriated via airlift to Germany. One cohort was triaged to the Cologne-Merheim Medical Center (Germany) for further medical care. We report on the tertiary medical care provided to this cohort of patients.

**Methods:**

This study is an observational report on complex wound management, infection and psychoemotional control associated with the 2004 Tsunami disaster. The setting was an adult intensive care unit (ICU) of a level I trauma center and subjects included severely injured tsunami victims repatriated from the disaster area (19 to 68 years old; 10 females and 7 males with unknown co-morbidities).

**Results:**

Multiple large flap lacerations (2 × 3 to 60 × 60 cm) at various body sites were characteristic. Lower extremities were mostly affected (88%), followed by upper extremities (29%), and head (18%). Two-thirds of patients presented with combined injuries to the thorax or fractures. Near-drowning involved the aspiration of immersion fluids, marine and soil debris into the respiratory tract and all patients displayed signs of pneumonitis and pneumonia upon arrival. Three patients presented with severe sinusitis. Microbiology identified a variety of common but also uncommon isolates that were often multi-resistant. Wound management included aggressive debridement together with vacuum-assisted closure in the interim between initial wound surgery and secondary closure. All patients received empiric anti-infective therapy using quinolones and clindamycin, later adapted to incoming results from microbiology and resistance patterns. This approach was effective in all but one patient who died due to severe fungal sepsis. All patients displayed severe signs of post-traumatic stress response.

**Conclusion:**

Individuals evacuated to our facility sustained traumatic injuries to head, chest, and limbs that were often contaminated with highly resistant bacteria. Transferred patients from disaster areas should be isolated until their microbial flora is identified as they may introduce new pathogens into an ICU. Successful wound management, including aggressive debridement combined with vacuum-assisted closure was effective. Initial anti-infective therapy using quinolones combined with clindamycin was a good first-line choice. Psychoemotional intervention alleviated severe post-traumatic stress response. For optimum treatment and care a multidisciplinary approach is mandatory.

## Introduction

Following the 2004 tsunami disaster that hit southeast Asia and killed over 225,000 people [1,2], severely injured tourists from various European countries were evacuated via airlift to Germany using German Air Force Airbus A310 MRT MedEvac transport [3-5]. Triage upon arrival at Cologne-Bonn Military Airport identified a cohort of 17 patients requiring further intensive medical care. This cohort was immediately transferred to the nearest level 1 trauma center of the region, the Cologne-Merheim Medical Center (CMMC). Rapid communication on different aspects associated with the long-distance air transfer, characteristic injury patterns, microbiological and psychoemotional findings at a very early stage following the disaster have previously been published [5,6]. The focus of the present report is given to tertiary medical care provided to this unique cohort of patients, in particular with respect to complex wound management, infection and psychoemotional control. According to the concept of a trimodal distribution of medical problems after large-scale disasters [7], the cohort evacuated to our facility had already entered the third phase of post-disaster medical care. During this phase (days to weeks after the tragic event) major efforts were undertaken to prevent and treat complications.

## Materials and methods

### Patients

Seventeen severely injured tsunami victims (19 to 68 years of age; 10 females and 7 males with unknown co-morbidities) needing further sophisticated medical care were immediately transferred to the CMMC (level 1 trauma center) following long distance tertiary air transfer and triage at Cologne-Bonn Military Airport. Detailed information on triage and initial care in the disaster region [8,9] and medical aspects associated with the airlift to Germany have been provided [5]. The patients arrived in our facility on average five days (three groups: range three to seven days) following the disaster. Upon arrival in our emergency department (ER), seven patients were intubated and mechanically ventilated and three patients needed catecholamines. All patients underwent standard clinical assessment and management as routinely performed on incoming patients, including rapid stabilization of vital parameters, physical and neurological examination, radiography and laboratory analysis. Patients on catecholamines upon arrival showed clinical and laboratory signs of severe sepsis [10].

### Complex wound management via vacuum-assisted closure therapy

Vacuum-assisted closure therapy (VAC Vakuumquellen, KCI Therapiegeräte, Höchstadt, Germany) was designed to promote the formation of granulation tissue in the wound bed, either as an adjunct to surgical therapy or as an alternative to surgery [11]. In detail, foam dressing with an attached evacuation tube is inserted into the wound and covered with an adhesive drape creating an airtight seal. Controlled, localized negative pressure is applied and effluents from wounds are collected into a nearby cannister. It is hypothesized that negative pressure contributes to wound healing by: (i) removing infectious materials and excess interstitial fluids, thus allowing tissue decompression [12]; (ii) increasing the vascularity of the wound, thus improving cutaneous perfusion [13,14]; (iii) promoting granulation tissue formation [15,16]; and/or (iv) creating beneficial mechanical forces that draw wound edges closer together. Vacuum-assisted wound closure may be considered medically necessary for patients with complicated surgical wounds when both of the following criteria are met: (i) need for accelerated formation of granulation tissue that cannot be achieved by other available topical wound treatments; and (ii) there is risk or co-morbidity present that is expected to significantly prolong healing achievable with other topical wound treatments [17]. A complicated surgical wound is a wound likely to take significantly longer to heal than a similar wound without complications, such as a large dehiscence or a significant wound infection.

### Microbiology

Surveillance cultures are a standard procedure in our facility when patients have been transferred or admitted from other areas or hospitals. Multiple and multifocal microbiological assessments were performed in each patient immediately upon arrival. Wound swabs, nasal swabs and respiratory tract specimens were cultured on the following agars: (i) Columbia 5% sheep blood; (ii) Mac Conkey; (iii) Chocolat+ PolyVite X (PVX) (Biomerieux, Nuertingen, Germany); (iv) Schaedler Kanamycin-Vancomycin 5% sheep blood (Becton Dickinson, Heidelberg, Germany); (v) Thioglycolat bouillon; and (vi) Sabouraud (Biomerieux, Nuertingen, Germany). Aerobic and anaerobic incubation, when appropriate for culture media, was performed at 35°C. Bacterial strains were identified using the Vitek 2 system and the API identification system (Biomerieux, Nuertingen, Germany). Antibiotic susceptibility was determined using the Vitek 2 system, disc-diffusion susceptibility testing and the E-Test (Ab Biodisk, Solna, Sweden). In those patients presenting with clinical signs of sepsis or who were highly suspicious for developing sepsis (*n *= 4), three sets of blood cultures were obtained immediately upon arrival and cultivated according to standard procedures and protocols.

### Psychological interventions

A severe degree of psychoemotional trauma was expected among all incoming patients and relatives and psychotherapeutic support was introduced as early as possible. The service was provided by the department's psychotherapeutic intervention team consisting of three qualified and experienced psychotraumatologists available 24 hours a day, 7 days a week upon request. Psychological services included psychoemotional support, intervention and counselling.

## Results

### Wound management

**Figure 1:**
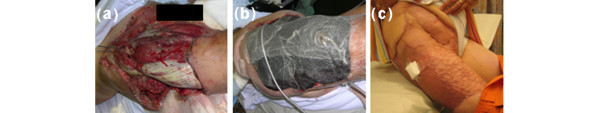
Wound management via vacuum-assisted closure therapy. **(a) **Large-scale tissue damage at hip and upper lower extremity. **(b) **Vacuum-assisted closure therapy. **(c) **Successful skin grafting.

**Figure 2:**
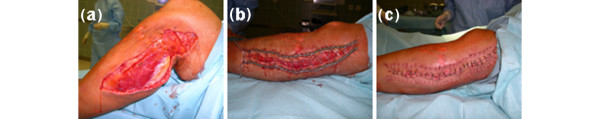
Wound management from primary surgery to delayed secondary closure. **(a) **Large-scale tissue damage at right lower extremity. **(b) **Cross-over technique for wound edge adaptation. **(c) **Definitive wound closure via suture.

**Figure 3:**
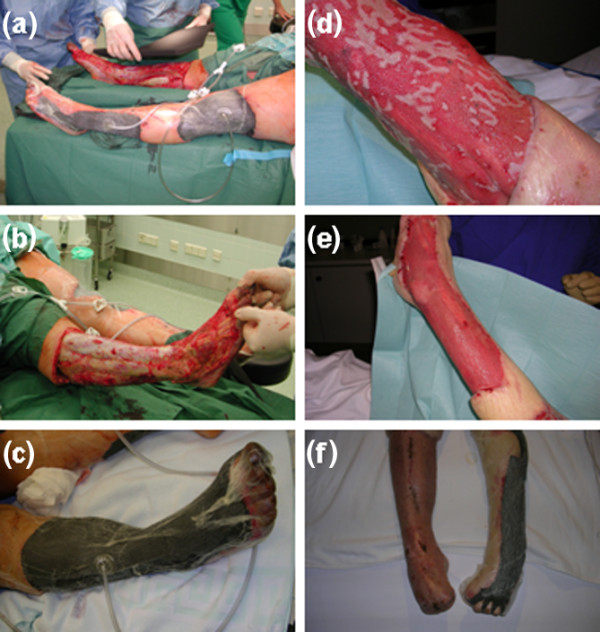
Wound management from primary surgery to delayed secondary closure. **(a-c) **Large-scale tissue damage at both lower extremities and vacuum sealing. **(d,e) **Wound site fills with granulation tissue. **(f) **Skin grafting at right lower extremity. Note that toe amputations had to be performed at right lower extremity due to severe septic microembolism.

Physical examination upon arrival at the ER revealed a pattern of severe large-scale soft-tissue damage common to 16/17 victims. Multiple large flap lacerations at various body sites were characteristic, ranging from 2 × 3 cm to 60 × 60 cm in size (Figures 1a, 2a and 3a, 3b). Lower extremities were mostly affected (88%), followed by upper extremities (29%), and head (18%). Two-thirds of patients had combined injuries to the thorax (for instance, pneumo-/hemopneumothorax), including intrapulmonary contusions and lesioning, and fractures of the extremities, both open and closed. Initial wound management focused on surgical removal of devitalized tissue and aggressive debridement. During the interim between initial wound surgery and secondary closure, wounds were protected using vacuum-assisted closure (Figures 1b and 3a, 3c, 3f). Renewal of vacuum-assisted wound dressings was performed in two to three day intervals under sterile conditions in the operating theatre. In two cases, amputations were inevitable due to septic microembolism resulting in severe acral necrosis (Figure 3f, left). Following conditioning (Figures 2b and 3d, 3e), wounds were closed either with or without skin grafting (Figures 1c, 2c and 3f).

### Infection control

#### Wounds

**Figure 4:**
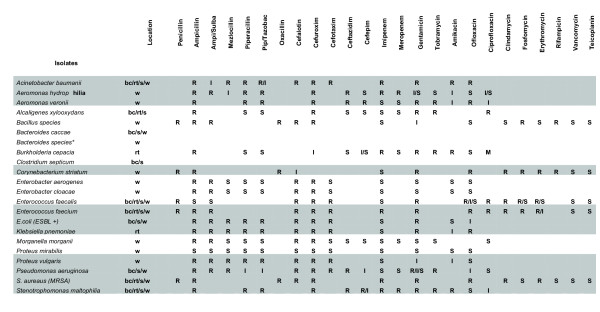
Resistance patterns for isolates from blood cultures, respiratory tracts, serum, and wounds. Isolates with multiple resistancies are in bold. ^a^Location: bc, blood culture; rt, respiratory tract; s, serum; w, wounds. ^b^*B. distasonis, fragilis, thetaiotaomicron*. ESBL, extended-spectrum β-lactamase; I, intermediate sensitive; R, resistant; S, sensitive.

Although wounds had already been cleaned and treated during the initial phase of care at primary medical facilities, all wounds were significantly contaminated with foreign material upon arrival of the patients in our facility (for example, with seawater, mud, sand, vegetation, corals, etc.). Cultures from repetitive wound swabs grew a variety of pathogens as summarized in Figure 4 and Table 1. Among those, a substantial number of highly resistant species was identified, including multiply resistant *Acinetobacter baumanii*, intermediate sensitive to ampicillin/sulbactam only, *Enterococcus faecium*, sensitive to glycopeptides only, extended-spectrum β-lactamase (ESBL) producing *Escherichia coli *and multi-resistant *Proteus vulgaris*, both sensitive to carbapenems, amikacin, and quinolones only, *Pseudomonas aeruginosa*, sensitive to carbapenems and tobramycin only, methicillin-resistant *Staphylococcus aureus *(*MRSA*), sensitive to fosfomycin, rifampicin, linezolid and glycopeptides only, and *Stenotrophomonas maltophilia*, sensitive to ofloxacin only. Polymicrobial wound contamination also included contamination with fungi (for instance, *Candida albicans *as well as *non-albicans *species), and moulds that were identified as *Mucor *species, *Fusarium solani *and *Aspergillus fumigatus*.

#### Respiratory tract

**Figure 5:**
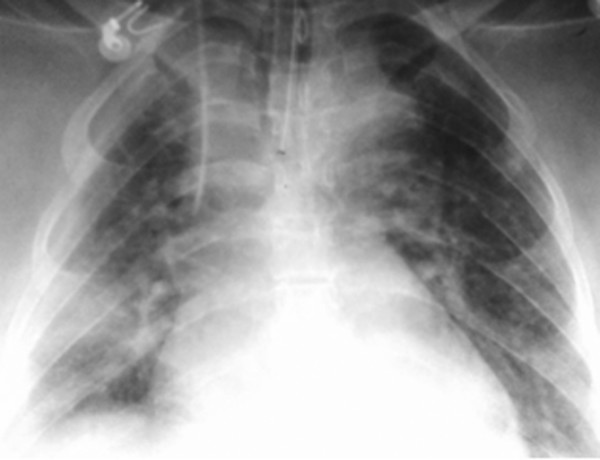
Chest radiography upon arrival displayed signs of pneumonia, for example, in the right lower lobe.

Tsunami near-drowning involved the aspiration of immersion fluids as well as marine and soil debris into the respiratory tract, thus producing intrapulmonary inoculation of bacteria. In accordance, all patients admitted to our facility displayed radiological and clinical signs of pneumonitis and pneumonia (Figure 5). Similar to wounds, microbiology from upper and lower respiratory tracts revealed a variety of common but also uncommon pathogens, including a substantial number of highly resistant species (Figure 4). For example, multiply resistant *A. baumanii *was isolated from respiratory tract specimens from all three patients that were in a septic state and required catecholamines upon ER arrival. Cultures further grew multiply resistant *E. faecium*, sensitive to glycopeptides only, *Klebsiella pneumoniae*, intermediate sensitive to amikacin only, *MRSA*, sensitive to fosfomycin, rifampicin, linezolid and glycopeptides only, and *Stenotrophomonas maltophilia*, sensitive to quinolones only.

#### Sinusitis

**Figure 6:**
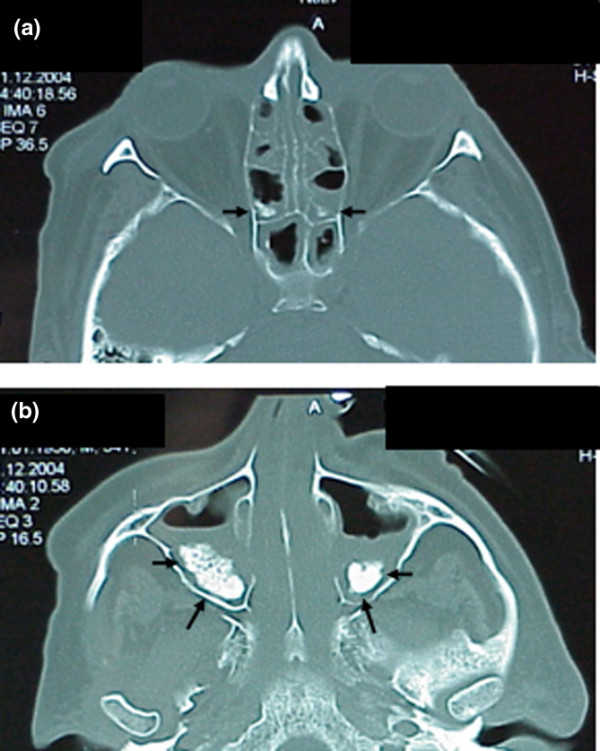
Computed cranial tomography (CCT): Arrows indicate fluid and opaque material in the **(a) **ethmoid and **(b) **maxillary sinuses.

Injuries associated with the tsunami disaster also involved sinusitis from inhaled seawater. Computed tomography from three patients showed fluid and opaque material in the ethmoid, maxillary, and sphenoid sinuses (Figure 6a, 6b) and purulent material and sand was removed via repeated washouts. Cultures from this material as well as from repeated nasal swabs grew multiply resistant *A. baumanii*, intermediate sensitive to ampicillin/sulbactam only, *E. faecium*, sensitive to glycopeptides only, and *C. albicans*. Cultures from nasal swabs from one patient were also highly suspicious for mould that was later identified as *Aspergillus fumigatus *(Table 1).

#### Systemic infection

Multiply resistant pathogens isolated from wounds, respiratory tracts and nasal swabs of three patients who arrived in a hemodynamically unstable condition had obviously triggered sepsis as these pathogens were also isolated from a series of blood cultures collected immediately upon ER arrival. Accordingly, blood cultures grew multiply resistant *A. baumanii*, intermediate sensitive to ampicillin/sulbactam only, *E. faecalis*, sensitive to ampicillin, carbapenemes, and glycopeptides only, *E. faecium*, sensitive to glycopeptides only, ESBL producing *E. coli*, sensitive to carbapenems, amikacin, and quinolones only, *MRSA*, sensitive to fosfomycin, rifampicin, linezolid and glycopeptides only, and *S. maltophilia*, sensitive to ofloxacin only (Figure 4).

#### Anti-infective therapy

All patients received empiric anti-infective therapy immediately upon arrival using a combination of quinolones and clindamycin. Anti-infective management was immediately adopted according to incoming results from microbiology and resistance patterns (Figure 4). Carbapenems and glycopeptides were frequently used within the later course to control infections involving multiply resistant *E. faecium *and *faecium*, *MRSA*, *Aeromonas *species, ESBL producing *E. coli*, *P. aeruginosa*, *K. pneumoniae*, and *S. maltophilia*. Attempts to control infection with multiply resistant *A. baumanii *involved sulbactam, if sensitive. In selected patients positive for MRSA, in which vancomycin was not effective, linezolid was applied. Fungal infections involving *C. albicans *as well as *non-albicans *species were successfully treated with voriconazole. Anti-infective treatment combined with consequent wound debridement and removal of devitalized tissues was effective in all but one patient. This patient was already highly septic on arrival at our facility, requiring high doses of catecholamines. He further presented with beginning renal and pulmonary failure. Microbiology from wounds, respiratory tract and blood cultures identified a high level of contamination with multiple multiply resistant pathogens, for example, *E. faecalis *and *faecium*, *C. albicans*, *F. solani*, *A. fumigatus*, *P. aeruginosa *and MRSA from wounds, *A. baumanii*, *Alcaligenes xylooxidans*, *E. faecalis *and *faecium*, *K. pneumoniae*, *MRSA* and *S. maltophilia *from the respiratory tract, *Candida *species and *E. faecium *from blood cultures, and *E. faecium *and *A. fumigatus *from nasal swabs. Within the later course, this patient developed severe fungal sepsis that could not be controlled. This patient died on day 32 following evacuation from the disaster area.

### Psychoemotional control

Among all patients and relatives, clinical symptoms of post-traumatic psychological stress response were noted. All patients treated in our hospital suffered at least loss of one relative, for example, a partner or child, and two mothers of our cohort lost both of their children. The majority of patients complained of nightmares, emotional detachment, sleep difficulties, flashbacks, headaches, and intrusive thoughts based upon their experiences during the disaster, such as awareness of people drowning and dying, or guilt and anxiety over children and relatives that were carried away by the wave and they were unable to save. Psychoemotional responses further comprised distress about injuries sustained, dissociation, optical, acoustical and olfactory intrusions and, in some cases, agitation.

## Discussion

We report on our experiences with respect to clinical wound management, infection control and psychoemotional trauma care in a cohort of German patients that were severely injured during the tsunami disaster in southeast Asia on 26 December 2004. These patients were initially stabilized in local medical facilities [8,9] and were then airlifted to the CMMC via German Air Force MedEvac Transport [5].

### Wound management

Deep and large flap lacerations at various body sites including significant tissue loss were the prominent pattern of injury in our cohort of victims repatriated from the disaster area. Similar injury characteristics have been reported by Leppaniemi and colleagues [6], who evacuated a second cohort of surviving tourists to Finland, and by Taylor and colleagues [7], who provided medical care after a series of tsunamis struck north Papua New Guinea in 1998. Injuries of that type require careful debridement including removal of devitalized and infected tissues while stabilizing remaining vital tissues, early operative care of critical structures to prevent later morbidity including amputation, and frequent wound dressing changes. These procedures are conceptually simple and common standard [18]. In the interim between surgery and secondary closure, with or without skin grafting, we demonstrate the effective use of vacuum-assisted closure systems. A major benefit associated with this approach is a reduced need for dressing changes that may be labor intensive and time consuming, in particular when providing critical care in the face of a large number of victims with significant soft tissue loss [19]. Further, vacuum-assisted closure therapy draws wounds closed by applying controlled, negative pressure while smoothly removing infectious material and interstitial fluids, thus allowing tissue decompression [12]. This promotes cutaneous perfusion [13,14] and formation of granulation tissue [15,16]. Using this approach, definitive wound closure could be achieved as early as within the first week following admission to our facility.

### Infection patterns

Traumatic wounds were immediately contaminated by a mixture of sea and fresh water, sewage, soil, foreign materials (for example, corals, sand, vegetation) and floating debris as many victims had been swept into the mangroves behind the shores by the force of the wave, causing polymicrobial infections [1,5]. Repeated multilocal microbiology identified a wide spectrum of bacteria common to the marine environment, for example, *Aeromonas *species [20]. Furthermore, the presence of enteric and Gram-negative pathogens/coliforms, for example, *E. coli *and *Proteus *and *Klebsiella *species, was not surprising as seawater is regularly contaminated with sewage, even in the best of times and that also in resort areas. Inland freshwater pools classically contain Gram-negative bacilli such as *Pseudomonas *species, *Aeromonas*, *Plesiomonas*, as well as *Burkholderia *and *Leptospira *[20-22]. Outbreaks of leptospirosis have been reported after flooding [23] but *Leptospira *was not isolated in our cohort. In contrast, *Aeromonas *and *Pseudomonas *species were frequently encountered in our cohort and have been associated with skin and soft tissue infections after traumatic exposure to contaminated water [22,24] as well as pulmonary complications and septicemia following near drowning [25-34]. Although atypical mycobacteria and anaerobic bacteria may also be encountered in wounds with fresh water or soil exposure [35], the most common pathogens associated with fresh water exposure remain staphylococci and streptococci [35]. *Burkholderia *species have only been anecdotally reported to induce necrotising pneumonia [36,37], cutaneous and septicaemic melioidosis [38-41].

Obviously, common hygiene standards could not be preserved during initial care in local settings due to the magnitude of the disaster, imposing limitations on the type and quality of services that could be provided. Thus, victims were additionally exposed to nosocomial pathogens. The disruption of clean water supplies was also a problem in local primary care settings and fecal contamination could be expected. While a variety of Gram-negative pathogens identified here presumably resulted from salt water immersion, others, such as *MRSA*, ESBL producing *E. coli*, *S. maltophilia *and *Enterococci*, could have come from water but were more likely acquired in triage facilities. Crowded conditions and limited sheltering may have facilitated the transmission of pathogens.

Interestingly, microbiology identified a range of highly resistant pathogens, notably multiply antibiotic-resistant *A. baumanii*. Severe infection due to multiply-resistant *A. baumanii *has also been reported in two tourists that were repatriated to Switzerland following the disaster [42]. It is known that *Acinetobacter *can survive on dry (for example, skin), and moist surfaces (for example, tracheobronchial tree). The environmental niche for this *Acinetobacter *is yet unknown, although it displays high antibiotic resistance when acquired in the environment [43]. To determine which of these organisms is causing infections and which are just colonizers is difficult.

Two patients developed severe systemic fungal infections due to *Mucor *and *Fusarium *species. Both species were isolated from multilocal wound specimens and swabs; in one patient, cultures additionally grew *A. fumigatus*. This patient did not survive. To date, one other patient with multifocal cutaneous mucormycosis complicating polymicrobial wound infection has been reported following the tsunami disaster [44]. In this case, histology confirmed the diagnosis and *Apophysomyces elegans *was isolated. The authors concluded that this patient most likely acquired mucormycosis from contamination of his wounds at the time of trauma or during first aid measures. Mucormycosis is caused by the *Mucor *mould species, which is a very common mould species readily found in soil, decaying vegetation, and water-damaged buildings worldwide and has previously but anecdotally been reported in wound infections from trauma [45], and natural disasters, for example, volcanic eruptions [46]. Fungal superinfection of wounds undoubtedly added substantially to the morbidity and mortality already recorded in tsunami-affected areas [42].

Sinusitis due to inhaled seawater during near drowning was not uncommon following the tsunami disaster. We report three cases and others have been reported [47] (Dr Jecker, University of Mainz Medical Center/Germany, personal communication). Cultures from our cohort grew multi-resistant *Acinetobacter*, *E. faecium*, mould and *Candida *species while Limchawalit and Suchato [47] described *Aeromonas *species, *Klebsiella*, *E. coli *and *Proteus mirabilis*. These pathogens were also identified from our cohort, although not from nasal specimens. Nasal swabs from three patients that were treated for acute sinusitis at the University of Mainz Medical Center (Germany) following the tsunami disaster grew *Plesiomonas shigelloides*, *Enteroccoci *and *P. mirabilis*. The occurrence of sinusitis associated with the tsunami disaster provides some estimation about the force with which the victims were hit and swept away by the wave.

### Antiinfective therapy

Our intial choice of anti-infective therapy was a combination of a potent quinolone combined with clindamycin. This strategy is commonly followed in our facility for infection of unknown origin and generally corresponds to the guidelines of the Paul-Ehrlich Society for Chemotherapy [48]. In addition, this approach covered major pathogens that could initially be expected in our incoming patients [35].

Quinolones, in particular those of group III, are effective against both Gram-positive and Gram-negative organisms. They further display excellent activity against *Enterobacteriaceae*, the enteric Gram-negative bacilli, including a variety of organisms resistant to penicillins, cephalosporins and aminoglycosides [48]. Quinolones have also been shown to have good activity against *Haemophilus influenzae*, penicillinase-producing *Neisseria gonorrhoe*, and *Campylobacter*. Of the Gram-postive organisms, staphylococci, including methicillin-resistant strains, are well inhibited, streptococci and pneumococci to a lesser extent. Inhibitory effects have been demonstrated against intracellular pathogens, for example, *Mycobacterium tuberculosis*, *Mycoplasma*, *Chlamydia*, *Legionella*, *Brucella *species, and *Pseudomonas *[48]. Therapeutic advantages associated with clindamycin include its wide distribution in all tissues, including bone and body fluids [48]. This was of particular interest as one out of four patients presented with open fractures and was thus at high risk for bone infection. Clindamycin further possesses an added virtue of excellent oral bio-availability. In post-disaster settings with reduced medical supplies, this may allow oral treatment to be virtually equivalent to parenteral therapy. Clindamycin has been shown to have good activity against staphylococci and streptococci, as well as anaerobic species, that is, *Bacteroides *species, *Corynebacteria*, and *Mycoplasma *[48].

### Psychoemotional aftermath

With respect to the tsunami's psychoemotional aftermath, the full impact of the wave on the mental health of the survivors is still unknown [2]. In February 2005 the World Health Organization, among others, estimated that up to 50% of the five million people affected by the tsunami would experience moderate to severe psychological distress. Approximately 5% to 10% would develop more persistent problems, for example, depression, post-traumatic stress disorder, or other anxiety disorders unlikely to resolve without intervention. The disaster may also have triggered acute episodes in cases of pre-existing conditions, in particular in patients that had been displaced from psychiatric facilities or that had lost their medication. The symptoms presented by our patients could be expected for the type of trauma sustained and included various forms of depression, post-traumatic stress disorder, characterized by flashbacks, emotional detachment, sleep difficulties, and other disruptions, and other anxiety disorders [2]. Psychological counseling and intervention was initiated as early as possible and led to relief of symptoms.

To cover the psychoemotional trauma that occurred with the disaster, non-governmental organizations and their local partners undertook all efforts to assure initial psychological support already at the scene. Upon arrival in Germany, psychological care continued directly at airports of arrival for those being evacuated by disaster intervention teams and emergency pastors, coordinated by NOAH (Nachsorge, Opfer- und Angehörigenhilfe), a special division of the Federal Office for Civil Protection and Disaster Management (Bundesamt für Bevölkerungsschutz und Katastophenhilfe). This network also introduced telephone hotlines, assembled passenger lists together with airline companies comprising less severely injured patients who were evacuated on regular flights, and distributed educational pamphlets on typical clinical signs of post-traumatic stress syndrome to each arriving victim, indicating when to consult professional support. Upon federal request the Department of Psychotraumatology of the University of Heidelberg (Germany) assembled a comprehensive list of 400 qualified psychotherapists offering immediate support nationwide when needed. These structures were not present prior to the 2004 tsunami disaster and it is intended to preserve and to further develop these structures and databases to be better prepared for future catastrophes. Thus, the foundation of a nationwide and independent Institute for Psychotraumatology has been discussed [49].

The area of disaster mental health is fairly new and only few data exist on what interventions may encounter short and long term psychological problems. One reason why valid epidemiological data are not yet sufficiently available may be related to the fact that most researchers felt that it would be unethical to perform investigations immediately after the disaster. A major challenge, for example, would be for upcoming epidemiological studies to differentiate normal stress and grief from psychopathological responses, and this in particular across cultural boundaries. For example, many health care providers that worked with local tsunami victims noted remarkable resilience. Obviously, Asian culture that puts strong emphasis on family and community ties and that puts group welfare over self-reliance appeared to have been a powerful tool in overcoming the disaster. Another point of discussion should be related to the overemphasis of finding and treating post-traumatic stress disorder. The importance of post-traumatic stress disorder in disaster mental health has been heavily debated over the past years as it may be assumed that other depressive and anxiety disorders apart from post-traumatic stress disorder may be overlooked, as might people with pre-existing conditions [2].

## Conclusion

Severe large scale soft-tissue damage, including high-level contamination, was common to all tsunami victims repatriated from the disaster area. During the interim between initial wound surgery and secondary closure, vacuum-assisted closure therapy was successfully used for wound protection and conditioning. Multilocal surveillance cultures identified a range of pathogens, some of which were highly antibiotic resistant. Transferred patients from disaster areas should be placed into contact and respiratory isolation until their microbial flora is identified as they present a threat for introducing new pathogens into an intensive care unit. Initial anti-infective therapy using quinolones combined with clindamycin appeared useful and a good first-line choice. Caregivers need to keep an open eye for the broad range of infectious processes that can cause febrile illnesses and local complications. Psychoemotional intervention successfully alleviated severe post-traumatic stress responses. Thus, for optimum treatment and care a multidisciplinary approach is mandatory.

## Key messages

• 	Individuals evacuated to our facility following the 2004 tsunami disaster sustained traumatic injuries to head, chest, and limbs that were often contaminated with highly resistant bacteria.

• 	Multiple large flap lacerations at various body sites were characteristic.

• 	Wound management including aggressive debridement together with vacuum-assisted closure in the interim between initial wound surgery and secondary closure was effective.

• 	Transferred patients from disaster areas should be isolated until their microbial flora is identified as they may introduce new pathogens into an intensive care unit.

• 	Psychoemotional intervention alleviated severe post-traumatic stress response.

## Abbreviations

CMMC = Cologne-Merheim Medical Center; ER = emergency department; ESBL = extended-spectrum β-lactamase; MRSA = methicillin-resistant *Staphylococcus aureus*.

## Competing interests

The authors declare that they have no competing interests.

## Authors' contributions

MM, SG, NY, FW continuously provided intensive care to the patients presented here. MM, SG, NY, CS, TP, DR, MMH, CR, SS, WP, BB carried out the surgical interventions on the patients presented here. AH provided detailed information on the radiology findings presented here. RS carried out the microbiological assessments. MM drafted the manuscript. All authors read and approved the final manuscript.

## Figures and Tables

**Table 1:** Yeast and mould species isolated from blood cultures, respiratory tracts, serum, and wounds

Isolate	Location
*Aspergillus fumigatus*	rt/w
*Candida albicans*	bc/rt/s/w
*Candida glabrata*	w
*Candida tropicalis*	bc/s/w
*Fusarium solani*	w
*Mucor *species	w

Bc, blood culture; rt, respiratory tract; s, serum; w, wounds.

## References

[B1] (2005). Rapid health response, assessment, and surveillance after a tsunami – Thailand, 2004–2005. MMWR Morb Mortal Wkly Rep.

[B2] Miller G (2005). The Tsunami's psychological aftermath. Science.

[B3] Gabel A (2005). Evakuierungsoperation in Asien: Luftbrücke für die Flutopfer. Dt Ärzteblatt.

[B4] Zylka-Menhorn V (2005). Bundeswehr-Airbus MedEvac: Ein Blick in die fliegende Intensivstation. Dt Ärzteblatt.

[B5] Maegele M, Gregor S, Steinhausen E, Bouillon B, Heiss MM, Perbix W, Wappler F, Rixen D, Geisen J, Berger-Schreck B, Schwartz R (2005). The long-distance tertiary air transfer and care of tsunami victims: injury pattern and microbiological and psychological aspects. Crit Care Med.

[B6] Leppaniemi A, Vuola J, Vornanen M (2005). Surgery in the air-evacuating Finnish tsunami victims from Thailand. Scand J Surg.

[B7] Taylor PRP, Emonson DL, Schlimmer JE (1998). Operation Shaddock: The Australian defense force response to the tsunami disaster in Papua New Guinea. Med J Aust.

[B8] Wattanawaitunechai C, Peacock SJ, Jitpratoom P (2005). Tsunami in Thailand -disaster management in a district hospital. N Engl J Med.

[B9] Kongsaengdao S, Bunnag S, Siriwiwattnakul N (2005). Treatment of survivors after the tsunami. N Engl J Med.

[B10] Bone RC, Balk RA, Cerra FB, Dellinger RP, Fein AM, Knaus WA, Schein RM, Sibbald WJ (1992). Definitions for sepsis and organ failure and guidelines for the use of innovative therapies in sepsis. The ACCP/SCCM Consensus Conference Committee. American College of Chest Physicians/Society of Critical Care Medicine. Chest.

[B11] Fleischmann W, Lang E, Russ M (1997). Infektbehandlung durch Vakuumversiegelung. Unfallchirurg.

[B12] Jukema GN, Böhm HJ, Hierholzer G (1997). Vakuumversiegelung: Ein neues Konzept zur Behandlung von Weichteil- und Knocheninfektionen. Langenbecks Arch Chir Suppl Kongresstol.

[B13] Mullner T, Mrkonjic L, Kwasny O, Wecsei V (1997). The use of negative pressure to promote the healing of Tissue defects: a clinical trial using the vacuum sealing technique. Br J Plast Surg.

[B14] Mendez-Eastman S (1998). Negative pressure wound therapy. Plast Surg Nurs.

[B15] Argenta LC, Morykwas MJ (1997). Vacuum assisted closure: A new method for wound control and treatment: Clinical experience. Ann Plast Surg.

[B16] Joseph E, Hamori CA, Bergman S, Roaf E, Swann NF, Anastasi GW (2000). A prospective randomized trial of vacuum assisted closure versus standard therapy of chronic nonhealing wounds. Wounds.

[B17] BCBSA Medical Policy Reference Manual. http://www.bcbsnc.com/services/medicapolicpdvacuum_assisted_closure_of_wounds.pdf.

[B18] Holian AC, Keith PP (1998). Orthopedic surgery after the Aitape tsunami. Med J Aust.

[B19] Driess D, Perry J (2005). Tsunami disaster: A report from the front. Crit Care Med.

[B20] Holmberg SD (1988). Vibrios and Aeromonas. Infect Dis Clin North Am.

[B21] Semel JD, Trenholme G (1990). Aeromonas hydrophilia water-associated traumatic wound infections: A review. J Trauma.

[B22] Gold WL, Salit IE (1993). Aeromonas hydrophilia infections of skin and soft tissue: Report of 11 cases and review. Clin Infect Dis.

[B23] Easton A (1999). Leptospirosis in Phlippine floods. BMJ.

[B24] Hiransuthikul N, Tantisiriwat W, Lertutsahakul K, Vibhagool A, Boonma P (2005). Skin and soft-tissue infections among tsunami survivors in southern Thailand. Clin Infect Dis.

[B25] Rosenthal SL, Zuger JH, Apollo E (1975). Respiratory colonization with pseudomonas putrefaciens after near-drowning in salt water. Am J Clin Pathol.

[B26] Reines HD, Cook FV (1981). Pneumonia and bacteremia due to Aeromonas hydrophilia. Chest.

[B27] Genoni L, Domenighetti G (1982). Near-drowning in an adult: favourable course after 20-minute submersion. Schweiz Med Wochenschr.

[B28] Sims JK, Enomoto PI, Frankel RI, Wong LM (1983). Marine bacteria complicating seawater near-drowning and marine wounds: a hypothesis. Ann Emerg Med.

[B29] Outin HD, Chatelin A, Ronco E, Nauciel C, Gajdos P, Barois A, Goulon M (1984). Aeromonas hydrophila septicemia: 3 cases, 1 with mediastinitis. Ann Med Interne.

[B30] Lee N, Wu JL, Lee CH, Tsai WC (1985). Pseudomonas pseudomallei infection from drowning: the first reported case in Taiwan. J Clin Microbiol.

[B31] Ender PT, Dolan MJ (1997). Pneumonia associated with near-drowning. Clin Infect Dis.

[B32] Ender PT, Dolan MJ, Dolan D, Farmer JC, Melcher GP (1996). Near-drowning-associated Aeromonas pneumonia. J Emerg Med.

[B33] Miyake M, Iga K, Izumi C, Miyagawa A, Kobashi Y, Konishi T (2000). Rapidly progressive pneumonia due to Aeromonas hydrophilia shortly after near-drowing. Intern Med.

[B34] Maluping RP, Lavilla-Pitogo CR, DePaola A, Janda JM, Krovacek K (2004). Occurrence, characterization and detection of potential virulence determinants of emerging aquatic bacterial pathogens from the Philippines and Thailand. New Microbiol.

[B35] Lim PL (2005). Wound infections in tsunami survivors: A commentary. Ann Acad Med Singapore.

[B36] Allworth AM (2005). Tsunami lung: a necrotising pneumonia in survivors of the Asian tsunami. Med J Aust.

[B37] Chierakul W, Winothai W, Wattanawaitunechai C, Wuthiekanum V, Rugtaengan T, Rattanalertnavee J, Jitpratoom P, Chaowagul W, Singhasivanon P, White NJ (2005). Melioidosis in 6 tsunami survivors in southern Thailand. Clin Infect Dis.

[B38] Maccanti O, Pardelli R, Tonziello A, Gracci G, Vivaldi I, Bolognesi L, Pantani C, Sani S (2004). Melioidosis in a traveller from Thailand: Case reposrt. J Chemother.

[B39] Wang YS, Wong CH, Kurup A (2003). Cutaneous melioidosis and necrotizing fasciitis caused by Burkholderia pseudomallei. Emerg Infect Dis.

[B40] Svensson E, Welinder-Olson C, Claesson BA, Studahl M (2006). Cutaneous melioidosis in a Swedish tourist after the tsunami in 2004. Scand J Infect Dis.

[B41] Nieminen T, Vaara M (2005). Burkholderia pseudomellei infections in Finish tourists injured by the December 2004 tsunami in Thailand. Eurosurveillance Weekly Archives.

[B42] Garzoni C, Emonet S, Legout L, Benedict R, Hoffmeyer P, Bernard L, Garbino J (2005). Atypical infections in tsunami survivors. Emerg Infect Dis.

[B43] Masur H, Murray P (2005). Tsunami disaster and infection: Beware what pathogens the transport delivers to your intensive care unit. Crit Care Med.

[B44] Andresen D, Donaldson A, Choo L, Knox A, Klaassen M, Ursic C, Vonthethoff L, Krilis S, Konecny P (2005). Multifocal cutaneous mucormycosis complicating polymicrobial wound infections in a tsunami survivor from Sri Lanka. Lancet.

[B45] Seguin P, Musellec H, Le Gall F, Chevrier S, Le Bouquin V, Malledant Y (1999). Post-traumatic course complicated by cutaneous infection with Absidia corymbifera. Eur J Clin Microbiol Infect Dis.

[B46] Patino JF, Castro D, Valencia A, Morales P (1991). Necrotizing soft tissue lesions after volcanic cataclysm. World J Surg.

[B47] Limchawalit K, Suchato C (2005). Images in clinical medicine. Tsunami sinusitis. N Engl J Med.

[B48] Bodmann KF, Vogel F (2001). Antimikrobielle Therapie der Sepsis. Empfehlungen einer Arbeitsgruppe der Paul-Ehrlich Gesellschaft für Chemotherapy e.V. Chemother J.

[B49] Bühring P (2005). Flutkatastophe in Südasien: Traumatisierung vorbeugen. Dt Ärzteblatt.

